# Identification of the *GST* Gene Family in *Reaumuria soongorica* and Its Response to Drought Stress

**DOI:** 10.3390/biology15080660

**Published:** 2026-04-21

**Authors:** Jun Zhao, Liying Ma, Weibo Du, Qianwen Song, Luna Xing, Wei Chen, Qingyan Zhao, Chunlei Zhen, Songsong Lu

**Affiliations:** 1 Forest College, Gansu Agricultural University, Lanzhou 730070, China; zhaoj@st.gsau.edu.cn (J.Z.);; 2 Key Laboratory of Evidence Science Techniques Research and Application of Gansu Province, Gansu University of Political Science and Law, Lanzhou 730070, China

**Keywords:** *GST*, drought, molecular evolution, *Reaumuria soongorica*

## Abstract

This study conducted a genome-wide identification and functional analysis of the glutathione S-transferase (*GST*) gene family in the xerophytic desert shrub *Reaumuria soongorica*. A total of 67 *GST* genes were identified, classified into seven subfamilies, including Phi and Tau, with family expansion primarily attributed to small-scale duplication events. The findings revealed that *ResoGST52*, a member of the Tau subfamily, serves as a core gene in drought response, exhibiting significant upregulation of 2.40-fold in leaves and 9.01-fold in roots under drought stress. Mechanistic investigations indicated that the expression of *ResoGST52* is likely directly regulated by the transcription factor ResoDof17, with specific hydrogen bonding interactions identified between the two. Co-expression network analysis further demonstrated that *ResoGST52* cooperates with key pathways such as plant hormone signaling, MAPK cascades, and glutathione metabolism to collectively respond to drought stress. Notably, evolutionary analysis revealed that ResoGST52 has undergone positive selection, with three positively selected sites identified. Among these, the p.Ala115Ser mutation increases the volume of the protein’s active site pocket, while the remaining mutations enhance surface hydrophobicity, thereby improving protein stability and catalytic efficiency under extreme drought conditions. In summary, this study not only systematically identifies the *GST* gene family in *R. soongorica* but also elucidates the central role of ResoGST52 in drought adaptation through multiple layers—from transcriptional regulation and co-expression networks to protein structural adaptive evolution—providing valuable candidate genes and theoretical insights for genetic improvement of drought tolerance in crops.

## Simple Summary

Drought is a major threat to global agricultural and forestry production. This study aimed to discover how the glutathione S-transferase of a highly drought-tolerant desert shrub, *Reaumuria soongorica*, adapts to drought stress, hoping to identify useful genes for breeding more drought-resistant crops. In this shrub, we discovered 67 *GST* genes. Among them, a gene named *ResoGST52* was the most important. It became significantly more active during drought, especially in the roots. We found that its activity is likely controlled by the transcription factor ResoDof17 and that it works in coordination with other genes involved in stress signaling and antioxidant defense. Importantly, the ResoGST52 protein itself has evolved advantageous modifications; its structure has been naturally altered to become more stable and efficient under harsh, dry conditions. In conclusion, this study identifies ResoGST52 as a key factor contributing to the remarkable drought tolerance of this desert plant. Understanding its role provides a valuable candidate gene and a scientific basis for future efforts to enhance the drought resistance of important agricultural plants.

## 1. Introduction

Drought stress is one of the most severe environmental factors limiting crop yield, and global warming further exacerbates the occurrence of extreme drought conditions [1]. Water plays a crucial role in various physiological processes of plants, including growth, development, and metabolism. Water deficit significantly impairs plant growth, development, and morphological establishment [2]. Therefore, exploring the molecular regulatory mechanisms underlying plant drought resistance and breeding drought-tolerant crop varieties are of paramount importance. Desert plants inhabiting extreme environments have developed unique stress adaptation mechanisms through long-term evolution, and the superior stress-resistant genes they harbor hold significant application value in the genetic improvement of crops and forage grasses [3]. Numerous studies have elucidated the adaptive mechanisms of desert plants in response to drought and applied their genetic resources to crop breeding. For example, the genetic resources of the desert plant *Zygophyllum xanthoxylum* have been utilized in alfalfa (*Medicago sativa*), significantly enhancing its tolerance to drought stress [4,5,6,7]. This demonstrates the feasibility and potential of translating extreme adaptation traits from desert species to crops.

*Reaumuria soongorica*, a xerophytic dwarf shrub belonging to the family Tamaricaceae and genus *Reaumuria*, is one of the most widely distributed zonal and azonal constructive species in the desert regions of China, representing an excellent model for studying drought adaptation due to its exceptional drought tolerance, salt tolerance, and vitality under extreme arid conditions [7,8,9]. In recent years, research on drought adaptation in *R. soongorica* has primarily focused on morphological and anatomical aspects. Studies have revealed that the leaves of *R. soongorica* have evolved into granular structures suited to arid environments, characterized by a hard texture and containing high levels of polysaccharides, polyphenols, and secondary metabolites [10]. Additionally, physiological studies have shown that *R. soongorica* adapts to stressful environments by reducing leaf water potential, improving water use efficiency, enhancing photosynthesis, developing specialized stomata, secreting large amounts of salt through salt glands on the leaves, accumulating abundant osmolytes such as sucrose, malate, and proline, and increasing enzymatic activities in the antioxidant system [11,12,13,14]. However, despite its remarkable phenotypic resilience, the genetic basis and molecular adaptation mechanisms of *R. soongorica* remain largely unexplored, particularly regarding genome-wide responses at the gene family level.

Glutathione S-transferases (GSTs; E.C.2.5.1.18) are a major class of detoxification enzymes that catalyze the conjugation of glutathione (GSH) to a wide range of electrophilic and cytotoxic compounds, playing a pivotal role in cellular antioxidant defense and xenobiotic metabolism [15]. Beyond detoxification, their importance in abiotic stress adaptation, particularly drought, is well-established. Under drought stress, the overproduction of reactive oxygen species (ROS) causes oxidative damage [16,17,18]. GSTs contribute to ROS scavenging directly by metabolizing lipid peroxides and other toxic byproducts. A substantial body of evidence from functional studies underscores the positive role of *GSTs* in drought tolerance. For instance, overexpression of a tau-class *LeGSTU2* from tomato enhanced osmotic stress tolerance in Arabidopsis [19]. Similarly, overexpression of *OsGSTU4* in rice improved the mutant’s resilience to oxidative and drought stress [20]. In crops like wheat and barley, the induction of specific GST isoenzymes under water deficit has been correlated with varietal drought resistance [21]. Crucially, heterologous expression of drought-induced GST genes from crops, such as *ScGSTF30* from sugarcane and *CsGSTU8* from tea plants, has been shown to confer significantly enhanced drought survival in transgenic Arabidopsis [22,23,24]. These consistent findings across species validate GSTs as key genes for engineering improved drought tolerance.

With advances in omics technologies, the publication of the *R. soongorica* genome has provided a foundation for elucidating its drought response mechanisms [8]. Therefore, this study integrates genomics, transcriptomics, and structural biology to perform a genome-wide identification of the *GST* gene family in *R. soongorica*, analyze their expression patterns under drought stress, investigate the potential transcriptional regulation and co-expression network of key candidate genes, and explore the adaptive evolution of key genes at the protein level. We expect that this study will provide valuable genetic resources and insights for understanding plant drought tolerance mechanisms.

## 2. Materials and Methods

### 2.1. Data Sources and Genome Annotation

The genomic data used in this study were obtained from the NCBI database (https://www.ncbi.nlm.nih.gov/ (accessed on 19 May 2025)). The GenBank accession numbers for *Reaumuria songarica*, *Tamarix chinensis*, *Spinacia oleracea*, *Beta vulgaris*, *Bougainvillea glabra*, and *Chenopodium quinoa* are GCA_040143545.1, GCA_030549775.1, GCF_020520425.1, GCF_026745355.1, GCA_045838725.1, and GCF_001683475.1, respectively. For the annotation of the *R. songarica*, *T. chinensis*, and *B. glabra* genomes, this study employed an RNA-Seq-guided automated annotation pipeline. First, RNA-Seq data were aligned to the respective genomes using HISAT2 (v2.1.0) [25]. Transcripts were then assembled using StringTie (v2.2.3) [26]. Gene prediction was performed with AUGUSTUS (v3.5.0) [27] using *A. thaliana* as a reference to generate annotation files, and annotation completeness was assessed using BUSCO (5.6.0). The transcriptomic data used for annotating *R. songarica*, *T. chinensis*, and *B. glabra* were obtained from the NCBI database under project accession numbers PRJNA1295811, PRJNA1201798, and PRJNA1288140, respectively.

### 2.2. Gene Family Identification

Identification of the *GST* gene family was performed using a combination of domain-based searches with hmmsearch (v3.3.2) [28] and sequence similarity searches using BLAST (v2.17.0). Specifically, GST sequences from *A. thaliana* were downloaded from the TAIR database (https://www.arabidopsis.org/ (accessed on 23 May 2025)). A hidden Markov model (HMM) profile for the *A. thaliana GST* gene family was constructed using hmmbuild and subsequently used to search against the genomes of *R. songarica* and its related species with hmmsearch. Sequences meeting the threshold (E-value < 0.05) were selected as preliminary candidates. A new HMM profile was then constructed from the search for the GST gene family of each respective species, followed by another round of hmmsearch within its own genome. The conserved domains of the positive sequences were validated using NCBI-CDD (https://www.ncbi.nlm.nih.gov/Structure/bwrpsb/bwrpsb.cgi (accessed on 28 May 2025)). Subsequently, a blast database was constructed using makeblastdb, and the positive sequences were aligned back to their respective genomes using blastn (E-value < 0.05) to verify sequence completeness and remove redundancies, resulting in the final set of candidate *GST* genes for each species. *GST* genes were named using the first two letters of each word in the species’ Latin name, followed by a number.

### 2.3. Phylogenetic Tree Construction, Gene Structure, Motif, and Synteny Analysis

Phylogenetic analysis of the GST gene families from *R. songarica*, *T. chinensis*, *S. oleracea*, and *B. vulgaris* was conducted using IQ-TREE (v 2.2.0) [29] based on the maximum likelihood method. Multiple sequence alignment of CDS sequences was performed using MAFFT (v7.505) [30]. The alignment results were then automatically trimmed using trimAl (v1.4.1) [31] to eliminate low-quality alignment regions. Finally, a maximum likelihood phylogenetic tree was constructed from the trimmed sequences using IQ-TREE. The best substitution model was selected using the -m MFP parameter, with the optimal nucleotide substitution model determined to be GTR + F + R5. Node support was assessed with 2000 bootstrap replicates and the approximate likelihood ratio test. Bootstrap-based NNI optimization was enabled to improve topological accuracy. The phylogenetic tree was visualized using ChiPlot.

Gene structure diagrams were generated using GSDS (v2.0) (https://gsds.gao-lab.org/ (accessed on 5 July 2025)) to visualize the exon-intron organization. Motif analysis was performed using MEME (Version 5.5.9, https://meme-suite.org/meme/tools/meme (accessed on 5 July 2025)), with conserved motifs having an e-value < 0.05 being identified as positive motifs. Synteny analysis was conducted using TBtools (v2.0) [32]. Whole-genome duplication (WGD) or segmental duplication events were inferred using MCScanX.

### 2.4. Material Treatment and Transcriptome Analysis

Transcriptomic data for *R. songarica* under drought stress were derived from previous experiments conducted by our research group. Mature seeds of *R. songarica* were collected from its natural habitat in northwestern China. After germination and nursery cultivation, uniformly sized seedlings were transplanted into pots containing homogenized loess soil. Before initiating drought treatments, plants were acclimatized for 2 weeks under well-watered conditions in a semi-open rainout shelter. Three soil moisture regimes were established: well-watered control (CK, 80 ± 5% of field capacity), moderate drought (G1, 50 ± 5% of field capacity), and severe drought (G2, 30 ± 5% of field capacity). Each treatment included six independent biological replicates. The soil water content was maintained within the target range by the gravimetric method, with watering performed daily at 7:00 PM to ensure consistent plant growth. Leaf and root samples for transcriptome sequencing were collected at the end of the 120-day treatment period. Total RNA was extracted from the collected leaf and root tissues using the TRIzol reagent (Invitrogen, USA) according to the manufacturer’s instructions. The concentration and purity of the extracted RNA were assessed using a NanoDrop spectrophotometer, and RNA integrity was verified by 1% agarose gel electrophoresis. High-quality RNA samples (A260/A280 ratio between 1.8 and 2.0) were used for subsequent library construction. Sequencing was performed on the Illumina HiSeq 4000 platform (Illumina, San Diego, CA, USA), generating 150 bp paired-end reads. The quality of the raw sequencing data was evaluated. The average Q20 and Q30 scores for all samples were above 97% and 93%, respectively, and the GC content was within the expected range, confirming the high quality and reliability of the sequencing output.

Raw paired-end Illumina sequencing reads were subjected to quality control and filtering using fastp (v1.0.1) [33] to improve data reliability. Filtering criteria included: (1) removal of reads containing adapter contamination; (2) exclusion of reads with more than 10% ambiguous nucleotides (N); and (3) removal of low-quality reads where over 50% of bases had a Phred quality score ≤ Q20. Gene expression levels were quantified using RSEM (v1.3.3). Clean reads from each sample were aligned to the unigene dataset using Bowtie2 [34] to obtain raw read counts and normalized expression estimates. Gene expression abundance is expressed as Fragments Per Kilobase of transcript per Million mapped reads (FPKM). To assess data quality and reproducibility among biological replicates, Pearson correlation coefficients were calculated based on the FPKM expression matrix of all genes. Samples showing a correlation coefficient (R) < 0.80 with other replicates were considered outliers and excluded from subsequent analyses. Differential expression analysis between drought treatments and the control was performed using the DESeq2 package(v1.42.0). Genes with an absolute value of log2 fold change (log2FC) ≥ 1 and a false discovery rate (FDR) < 0.05 were considered significantly differentially expressed.

### 2.5. Transcriptional Regulatory Analysis Under Drought Stress

Expression profiles of *ResoGST* genes were visualized using the R package ComplexHeatmap (version 2.12.0) [35]. Volcano plots for differentially expressed genes were generated using the Python package pandas (version 0.23.4) [36]. Transcription factor binding sites were predicted using PlantTFDB (https://planttfdb.gao-lab.org/ (10 June 2025)) with a screening threshold of *p* < 0.05. Transcriptional regulatory networks were constructed and visualized using Cytoscape (v3.10.3) [37]. The conformational structure of transcription factor binding to ResoGST promoters was modeled using AlphaFold3 (https://alphafoldserver.com/ (accessed on 10 June 2025)) [38], and hydrogen bonds involved in the binding were analyzed using PyMOL (v2.0). Weighted Gene Co-expression Network Analysis (WGCNA) was performed using the R package WGCNA (version 1.71). To construct a scale-free co-expression network, an appropriate soft-thresholding power was determined. The analysis of network topology (Figure S2) indicated that a power of β = 18 was selected, as it was the lowest power for which the scale-free topology fit index (signed R^2^) reached above 0.8. A signed hybrid network was then built using this power with a minimum module size of 20 genes. Module detection was performed using the dynamic tree cut method with a deepSplit value of 2 and a mergeCutHeight of 0.25. KEGG pathway annotation and visualization were conducted using the R package ggplot2 (v 3.3.0).

### 2.6. Evolutionary Analysis and Protein Structure Comparison

Orthologous genes of ResoGST were identified using OrthoFinder (v2.5.5) [19]. Positive selection sites in ResoGST52 were detected using the branch-site model in PAML (v4.9) [39]. For branch-site analysis, model A0 and model A1 were compared, with significance determined by LRTs. Potential positive selection sites under the alternative models were identified using the Bayes Empirical Bayes (BEB) method, with sites having a posterior probability > 0.8 considered to be under significant positive selection. Protein models for ResoGST52 and SpolGST21 were constructed using AlphaFold3. Binding pockets were predicted using Proteins Plus (https://proteins.plus/ (accessed on 12 September 2025)) with the DoGSiteScorer algorithm, and pocket visualization was performed using molviz (https://molviz.com/ (accessed on 13 September 2025)). Protein structure comparisons were conducted using PyMOL. Atomic distances were measured using VMD (v2.0) [20]. The hydrophilicity of amino acid residues was analyzed using ExPASy (https://web.expasy.org/protscale/ (accessed on 15 September 2025)).

## 3. Results

### 3.1. Gene Identification

**Figure 1:**
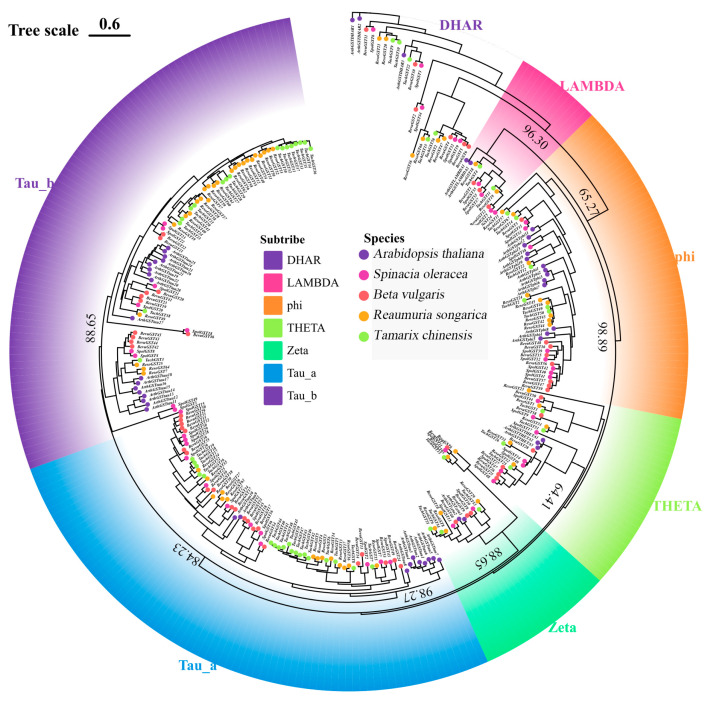
Phylogenetic tree of the *GST* gene family in *R. soongorica* and its closely related species. Numbers adjacent to nodes indicate the support values for the respective nodes.

The results of gene identification (Table S1) reveal that the *R. soongorica* genome contains 67 *GST* genes. In comparison, the genomes of several closely related species—Tamarix chinensis, Spinacia oleracea, and Beta vulgaris—contain 57, 49, and 59 *GST* genes, respectively, indicating that the evolutionary expansion of the *GST* gene family is not conserved in terms of gene number. A combined dataset of 283 GST genes from all species included in the phylogenetic analysis (containing 51 *GST* genes from *A. thaliana*) was constructed for subsequent evolutionary study. Phylogenetic analysis (Figure 1) demonstrates that *ResoGSTs* are divided into seven subfamilies: *DHAR*, *LAMBDA*, *Phi*, *THETA*, *Zeta*, *Tau_a*, and *Tau_b*. Each subfamily includes *GST* genes from *A. thaliana* and other closely related species, suggesting functional conservation of *GST* genes across evolution. Notably, *Tau_a* and *Tau_b* are the two largest subfamilies, containing 67/283 and 81/283 *GST* genes, respectively, with *R. soongorica* contributing 14 and 25 *GST* genes to these clades. The LAMBDA subfamily is the smallest, comprising 12 *GST* genes, including 2 *ArthGSTs*, 2 *ResoGSTs*, 2 *TachGSTs*, 3 *SpolGSTs*, and 3 *BevuGSTs*.

Physicochemical property analysis (Table S2) shows that ResoGSTs consist of 57–534 amino acids, with molecular weights ranging from 6563.64 to 59,941.26 Da and isoelectric points (pI) between 4.88 and 11.42. Among the ResoGSTs, only three are hydrophobic proteins (GRAVY > 0), while the majority are hydrophilic. Notably, only three ResoGSTs were predicted to be stable proteins based on the instability index, while the remainder were predicted as unstable.

### 3.2. Gene Structure of GSTs in R. soongorica

**Figure 2:**
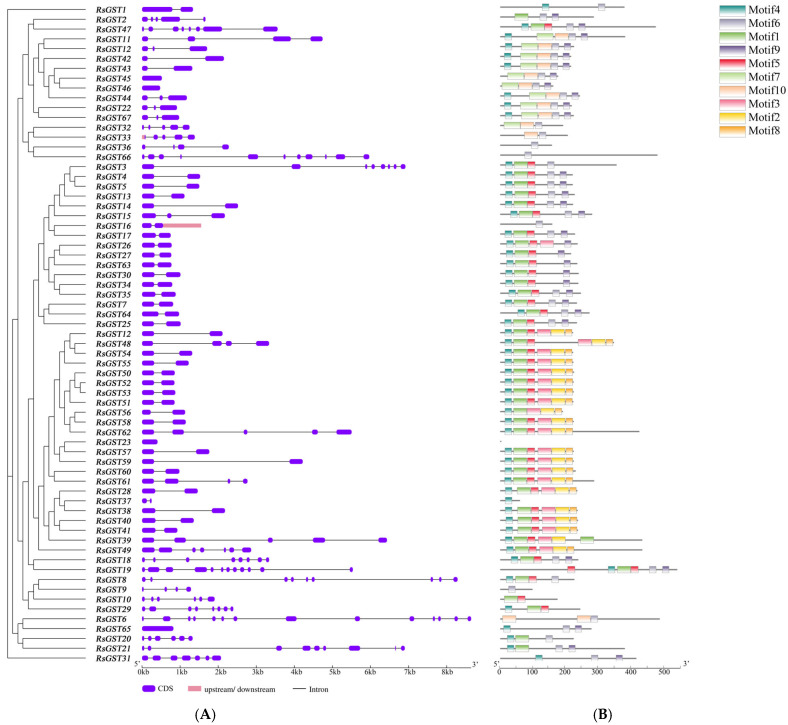
Schematic representation of gene structure and motif composition of *ResoGSTs*. (**A**) Gene structure of *ResoGSTs*. (**B**) Motif distribution of ResoGSTs.

Gene structure analysis (Figure 2) revealed that the full-length *ResoGST* genes range from 477 to 8643 Kb, with exon numbers varying from 1 to 14. Among these, *ResoGST6* is the longest, spanning 8643 Kb and containing 14 exons, while ResoGST46 is the shortest at 477 Kb with only one exon. The most prevalent structural type consists of 2 exons and 1 intron, observed in 33 *ResoGSTs*. A total of 10 distinct motifs were identified in *ResoGSTs*, with the number and type of motifs differing across subfamilies. ResoGST36, ResoGST9, and ResoGST37 each contain only one motif, whereas the majority of ResoGSTs possess six motifs, suggesting potential functional divergence among subfamilies.

### 3.3. Synteny Analysis of the GST Gene Family in R. soongorica

**Figure 3:**
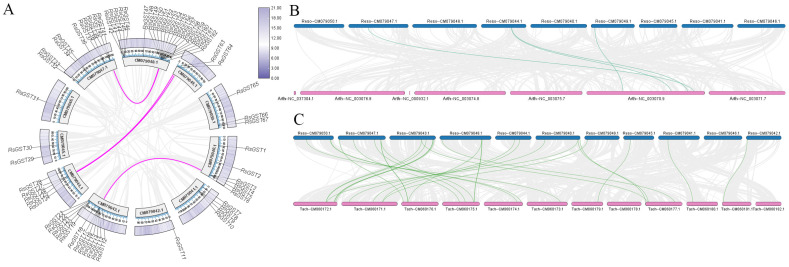
Synteny analysis of *ResoGSTs* within and across species. (**A**) Intra-species synteny of *ResoGSTs*. (**B**) Synteny between *ResoGSTs* and *ArthGSTs*. (**C**) Synteny between *ResoGSTs* and *TachGSTs*.

Chromosomal localization and intra-species synteny analysis (Figure 3A) revealed that *ResoGSTs* are distributed across 11 chromosomes, with the highest number (22 *ResoGSTs*) located on CM079048. In contrast, only one *ResoGST* was found on each of CM079046 and CM079042. Analysis of gene duplication mechanisms classified the 67 *ResoGST* genes into four types based on their origins: dispersed (23 genes, 34.3%), proximal (21 genes, 31.3%), tandem (13 genes, 19.4%), and WGD/segmental duplication (10 genes, 14.9%) (Figure S1). The fact that all genes could be classified confirms that duplication is the sole source for the expansion of this gene family. Notably, only four homologous gene pairs were identified via synteny analysis, consistent with the low proportion (14.9%) of genes derived from WGD/segmental events. This suggests that small-scale duplications (dispersed and proximal), rather than large-scale segmental duplication, are the primary mechanisms driving the expansion of this gene family. Inter-species synteny analysis (Figure 3B,C) indicated that *ResoGSTs* share four homologous gene pairs with *ArthGSTs* and 27 with *TachGSTs*. These findings imply a closer evolutionary relationship between *R. soongorica* and *T. chinensis*, while also highlighting potential functional divergence of *GST* genes across different lineages.

### 3.4. Transcriptional Regulation Patterns of ResoGSTs Under Drought Stress

**Figure 4:**
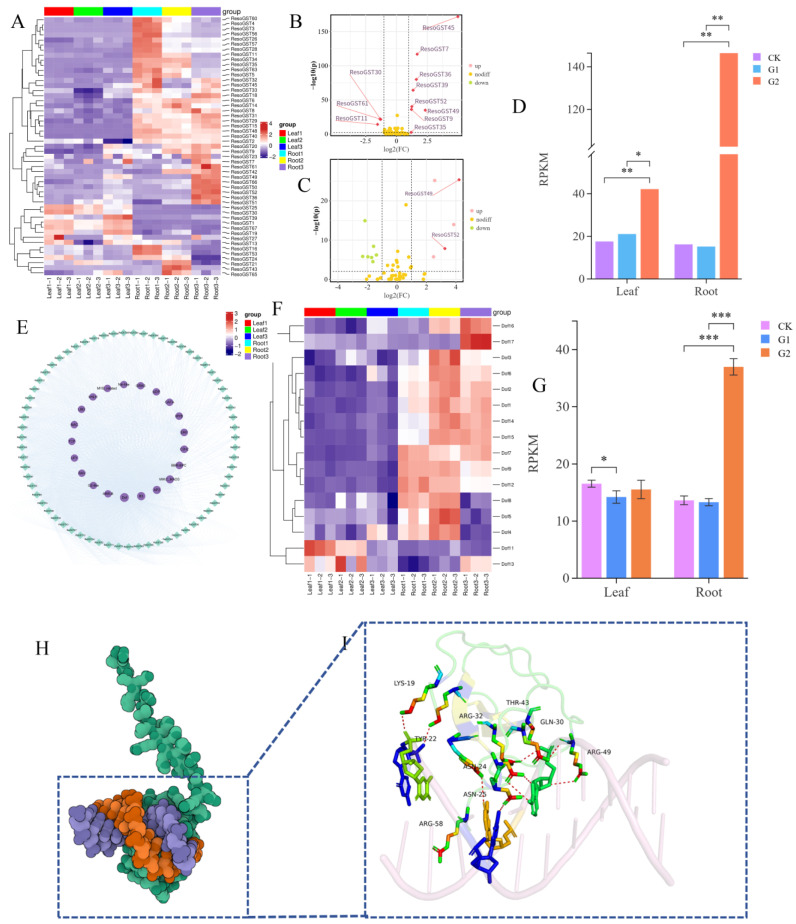
Transcriptional regulation analysis of *ResoGSTs* under drought stress. (**A**) Expression profiles of *ResoGSTs* under drought stress. (**B**) Differentially expressed *ResoGSTs* in roots. (**C**) Differentially expressed *ResoGSTs* in leaves. (**D**) Expression levels of *ResoGST52* under drought stress. (**E**) Regulatory network of transcription factors targeting *ResoGSTs*. (**F**) Expression profiles of *ResoDofs* under drought stress. (**G**) Expression levels of *ResoDof17* under drought stress. (**H**) Binding model of ResoDof17 to the promoter region of *ResoGST52*. (**I**) Hydrogen bond distribution at the binding interface between ResoDof17 and the promoter region of *ResoGST52*. * *p* < 0.05, ** *p* < 0.01, *** *p* < 0.001.

Expression profiling analysis (Figure 4A) revealed that *ResoGSTs* exhibited higher expression levels in roots compared to leaves. Under drought stress, most *ResoGSTs* showed no significant changes in expression in leaves, suggesting constitutive expression in this tissue. In contrast, the majority of *ResoGSTs* in roots displayed differential expression following drought stress. Differential expression analysis (Figure 4B) identified 11 *ResoGSTs* with altered expression in roots under drought conditions, including eight significantly upregulated and three significantly downregulated genes. In leaves, 12 *ResoGSTs* were differentially expressed; however, it is noteworthy that only *ResoGST49* and *ResoGST52* exhibited FPKM values greater than 5 (Figure 4C). These results suggest that *ResoGST52* may be a key *GST* gene involved in the drought stress response in *R. soongorica*, as it was significantly upregulated in both leaves and roots—by 2.40-fold in leaves and 9.01-fold in roots.

**Figure 5:**
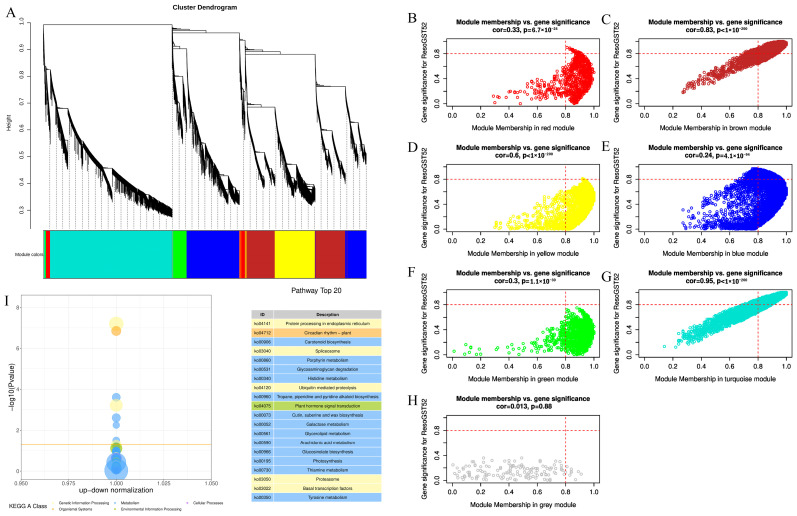
Co-expression module analysis of *ResoGST52* under drought stress. (**A**) Division of co-expression modules. (**B**) Correlation between the red module and *ResoGST52* expression levels. (**C**) Correlation between the brown module and *ResoGST52* expression levels. (**D**) Correlation between the yellow module and *ResoGST52* expression levels. (**E**) Correlation between the blue module and *ResoGST52* expression levels. (**F**) Correlation between the green module and *ResoGST52* expression levels. (**G**) Correlation between the turquoise module and *ResoGST52* expression levels. (**H**) Correlation between the gray module and *ResoGST52* expression levels. (**I**) KEGG enrichment pathways of co-expressed differentially expressed genes.

To investigate whether *ResoGSTs* are regulated by transcription factors, promoter sequences spanning 2000 bp upstream were extracted and analyzed for predicted transcription factor binding sites. The results (Figure 4E) indicated that *ResoGSTs* could be regulated by 21 transcription factor families, with Dof binding sites being the most abundant. Expression analysis of *ResoDofs* (Figure 4F) showed patterns similar to those of *ResoGSTs*, with higher expression levels observed in roots. Among these, *ResoDof17* exhibited the most pronounced expression difference, being upregulated by 2.29-fold in roots under drought stress (Figure 4G). To predict the potential binding capacity of Dof transcription factors to the promoter of *ResoGST52*, a structural model of their interaction was constructed (Figure 5H,I). The results demonstrated that the ResoDof17 protein specifically binds to the promoter sequence 5′-CTCAATTGTTT-3′ of *ResoGST52*, forming eight hydrogen bonds, providing a structural model for a potential regulatory relationship, which awaits experimental validation.

### 3.5. Co-Expression Analysis of R. soongorica GSTs Under Drought Stress

To elucidate the molecular regulatory mechanism of *ResoGST52* in *R. soongorica* under drought stress, this study used the expression data of *ResoGST52* from the drought-stressed transcriptome as a phenotypic indicator. WGCNA was performed on the root transcriptome data, and KEGG pathway enrichment analysis was conducted on the core co-expression module to identify the key metabolic pathways involved. Based on WGCNA, all expressed genes were clustered into seven co-expression modules according to expression pattern correlations (Figure 5A). Among these, the Turquoise module showed a high correlation with the expression pattern of *ResoGST52* (cor = 0.95, Figure 5G). This module contained 3135 differentially expressed genes, suggesting that these genes co-express with *ResoGST52* during the drought response of *R. soongorica* and may jointly participate in the molecular regulatory network of drought stress. To clarify the functional direction of the core co-expression module, KEGG pathway enrichment analysis was performed on the genes within this module (Figure 5I). A total of 154 pathways were enriched, with the main enrichment concentrated in the following core categories: stress signal transduction pathways, such as plant hormone signal transduction (ko04075) and MAPK signaling pathway in plants (ko04016), and secondary metabolite metabolic pathways, such as biosynthesis of amino acids (ko01230), starch and sucrose metabolism (ko00500), and phenylpropanoid biosynthesis (ko00940). Notably, pathways including ascorbate and aldarate metabolism (ko00053) and glutathione metabolism (ko00480) were also enriched, indicating that *ResoGST52* and its co-expressed genes likely enhance drought tolerance in *R. soongorica* by regulating the ascorbate-glutathione cycle to scavenge toxic substances such as reactive oxygen species induced by drought.

### 3.6. Evolutionary Analysis of ResoGST52 Protein

**Figure 6:**
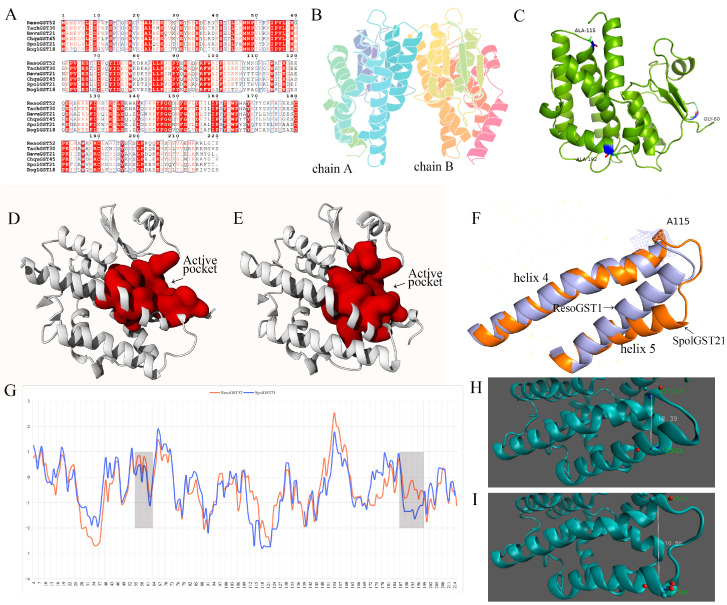
Molecular evolutionary analysis and protein structural comparison of ResoGST52. (**A**) Amino acid sequence alignment of ResoGST52 and its orthologous proteins. (**B**) Homodimeric structure of ResoGST52. (**C**) Locations of the four amino acid mutation sites on the ResoGST52 protein structure. (**D**) Schematic representation of the active pocket in SpolGST21. (**E**) Schematic representation of the active pocket in ResoGST52. (**F**) Positions of helix 4 and helix 5 in ResoGST52 and SpolGST21 proteins. (**G**) Hydrophilicity distribution of amino acid residues in ResoGST52 and SpolGST21. (**H**) Distance between helix 4 and helix 5 in the ResoGST52 protein. (**I**) Distance between helix 4 and helix 5 in SpolGST21 protein. Å denotes the unit of length (1 Å = 0.1 nm).

Evolutionary analysis revealed three significant positive selection sites in ResoGST52: p.Gly60Asn, p.Ala115Ser, and p.Ala192Glu, with Bayesian empirical Bayes (BEB) posterior probabilities of 0.959, 0.890, and 0.804, respectively (Table S3). The protein structure of ResoGST52 is a homodimer (Figure 6B). Among the identified sites, p.Gly60Asn and p.Ala192Glu are located on the protein surface, while p.Ala115Ser resides within the ligand-binding pocket (Figure 6C). Pocket prediction results (Figure 6D,E) showed that the active pocket volume of ResoGST52 is 1056.26 Å^3^, whereas that of SpolGST21 is 850.11 Å^3^, suggesting that the site mutations may have contributed to the enlargement of the active pocket in ResoGST52. Further investigation revealed that the p.Ala115Ser mutation reduced the distance between helix 4 and helix 5. The distance between residues 115 and 123 in ResoGST52 is 12.39 Å, compared to 16.86 Å in BesuGST21, which may explain the expansion of the active pocket in ResoGST52. Additionally, the mutations p.Gly60Asn and p.Ala192Glu increased the hydrophobicity in their respective regions, potentially enhancing the stability of ResoGST52 and enabling it to maintain catalytic activity under extreme environmental conditions.

## 4. Discussion

### 4.1. Genomic Landscape and Evolutionary Dynamics of the GST Gene Family in R. soongorica

The genome-wide identification of the *GST* gene family has been widely reported in many plant species. In this study, we identified 67 and 59 *GST* genes in the genomes of the desert shrubs *R. soongorica* and *T. chinensis*, respectively. Although this number is generally lower than that in many herbaceous plants. For example, the genomes of tomato, tea plant, barley, *Capsicum annuum*, *Setaria italica*, and *Prunus avium* contain 90, 88, 84, 85, 73, and 67 *GST* genes, respectively [40,41,42,43,44,45,46]. This variation is not strictly related to whether the plant is woody or herbaceous, as evidenced by the 49 *GST* genes found in spinach. Furthermore, these findings indicate that *GST* genes may have undergone gene family expansion or contraction events across different evolutionary lineages, leading to variations in the number of *GST* family members among plant species.

Generally, plant *GSTs* are classified into several subfamilies based on conserved active site residues, protein sequence similarity, and phylogenetic relationships, with *Phi*, *Tau*, *Lambda*, and *DHAR* being the major subfamilies [47]. Based on the clustering of *GSTs* from *R. soongorica* and its closely related species, the *GST* gene family of *R. soongorica* was divided into seven subfamilies, consistent with findings in other species. *Phi* and *Tau* were the subfamilies containing the largest number of *GST* members. Additionally, each subfamily included *GST* genes from *A. thaliana* and other closely related species, indicating that *GST* genes are relatively conserved in functional evolution.

In *R. soongorica*, the expansion of the *GST* gene family was primarily driven by small-scale duplication events, with dispersed, proximal, and tandem duplications collectively playing a dominant role. This contrasts with species such as apple, in which tandem duplication is considered the main driver of family expansion [41]. The prevalence of small-scale duplications in *R. soongorica* may have facilitated a more decentralized and flexible pattern of functional diversification. The duplicated genes can undergo subfunctionalization or neofunctionalization, thereby refining their spatial expression patterns or acquiring novel substrate specificities, which would fine-tune the plant’s adaptive responses to multifaceted desert stresses. Further supporting the theme of functional diversification, we observed considerable variation in gene structure among different *GST* subfamilies in *R. soongorica*, although members within the same subfamily share largely similar structures. A similar pattern has been reported in apples and sweet cherries [41,48], indicating that gene structure evolution represents a critical layer of *GST* family differentiation. The gain or loss of introns/exons potentially arising from chromosomal rearrangements [49] can influence mRNA processing, stability, and alternative splicing [47]. Consequently, the distinct gene architectures of *R. soongorica* GST subfamilies may underlie the generation of protein diversity and regulate expression dynamics, ultimately contributing to the complex regulatory networks required for adaptation to its harsh and variable habitat.

### 4.2. Members of the Core Tau Subfamily in Drought Response and Their Potential Regulatory Role

Drought is one of the most prevalent abiotic stresses, severely affecting plant growth and accelerating desertification. GSTs represent an intrinsic natural defense mechanism in plants, with members of the Tau subfamily playing a particularly significant role in enhancing drought tolerance. In Arabidopsis, overexpression of *LeGSTU2* alleviated osmotic stress responses induced by mannitol [50]. Additional studies suggest that *LeGSTU2* may function as a stress modulator by increasing SOD and POD activities, enhancing ROS scavenging capacity, or maintaining ROS homeostasis [51]. Moreover, overexpression of a tau-type *GST* gene improved mannitol stress resistance in transgenic tobacco. Overexpression of the tau-class *GST* gene *OsGSTU4* in Arabidopsis enhanced the mutant’s tolerance to oxidative stress [52]. Overexpression of *CsGSTU8* from the tea plant significantly improved drought tolerance in Arabidopsis mutants, manifested as enhanced scavenging of excess ROS under drought conditions [53]. Overexpression of the Tau subfamily member *ScGSTF30* from sugarcane markedly increased drought tolerance in transgenic Arabidopsis, primarily by enhancing osmoprotectant accumulation, activating antioxidant defenses, and reducing ROS damage, thereby positively regulating drought tolerance [54].

This study found that most *ResoGSTs* exhibited low expression levels, particularly in leaves, where expression was significantly lower than in roots, with the majority showing constitutive expression in leaves. Research in Nicotiana tabacum revealed significant differences in drought response patterns among different subfamily members, along with tissue-specific expression [24]. This indicates that the response of *GSTs* to drought stress is a complex process, likely involving diverse regulatory mechanisms.

Furthermore, this study identified *ResoGST52* as significantly upregulated in both leaves and roots under drought stress. Its expression under normal physiological conditions was relatively higher compared to other *ResoGSTs*. As *ResoGST52* belongs to the Tau subfamily, we hypothesize that it may be a key gene within the *ResoGST* family responding to drought stress. Co-expression analysis revealed that ResoGST52 is co-expressed with genes involved in plant hormone signal transduction, MAPK signaling, and glutathione and ascorbate biosynthesis. Plant hormones and MAPK phosphorylation cascade signaling, particularly ABA-mediated signaling transduction, serve as the primary signaling pathways in plant responses to drought stress [2]. Furthermore, glutathione and ascorbate are classic antioxidants that play a crucial role in coordinated oxidative stress management following drought exposure [16]. Therefore, we speculate that *ResoGST52* may exert protective effects through the antioxidant system.

Transcription factors serve as regulators enabling plants to adapt to environmental changes [55]. Dof transcription factors have been demonstrated to play transcriptional regulatory roles in plant responses to drought stress [36,56,57]. This study identified a substantial number of Dof binding sites in the promoter regions of ResoGSTs and found that some ResoDof members were differentially expressed under drought stress, particularly ResoDof17. Similarly, in the tea plant, *CsGSTU7* was found to potentially be regulated by Dof transcription factors [56]. Consequently, we hypothesize that the expression of *ResoGST52* may be transcriptionally regulated by ResoDof17.

### 4.3. Positive Selection of ResoGST52 Drives Adaptive Mutation to Enhance Its Function

Non-synonymous mutations modulate changes in protein function, facilitating the divergence of new genes and proteins. Therefore, exploring natural genetic variation is significant for enriching the genetic resource pool [58]. The significant positive selection pressure detected on ResoGST52 provides compelling molecular evidence for its role in the adaptation of *R. soongorica* to extreme arid environments. This selective force likely acts on specific non-synonymous mutations that fine-tune protein function, thereby enhancing plant fitness under drought stress. We identified three positive selection sites in the ResoGST52 coding sequence, each contributing to structural optimizations crucial for survival under water-limited conditions.

The p.Ala115Ser mutation, located near the substrate-binding pocket, is predicted to enlarge the active site cavity. This structural change holds significant adaptive value. As noted for other GSTs, a larger binding pocket can increase substrate promiscuity and affinity for the larger or diverse oxidative metabolites generated under severe drought [59]. This enhanced catalytic flexibility may allow ResoGST52 to neutralize a broader spectrum of drought-induced reactive electrophilic toxins more efficiently, providing a metabolic advantage.

Concurrently, the p.Gly60Asn and p.Ala192Glu mutations are predicted to primarily enhance protein stability. Under extreme water deficit, cellular conditions severely challenge protein folding and integrity, often leading to denaturation and loss of function [60]. The introduction of asparagine and glutamate at these positions is expected to strengthen intramolecular interactions, thereby rigidifying the protein structure. This increased stability ensures that ResoGST52 maintains its conformational integrity and catalytic activity under the harsh dehydration conditions typical of the plant’s natural habitat.

Collectively, these mutations illustrate a clear adaptive trajectory: natural selection in an extremely arid environment has shaped the ResoGST52 sequence to simultaneously optimize catalytic efficiency and preserve functional resilience. This dual optimization enables the enzyme to serve as a robust and versatile component of the antioxidant system, which may be highly beneficial for the adaptation of *R. soongorica* to extreme arid environments.

## 5. Conclusions

This study conducted a systematic identification and analysis of the *GST* gene family in the stress-tolerant plant *R. soongorica*. A total of 67 GST genes were identified, and the expansion of this gene family was found to be primarily driven by small-scale duplication events. The research identified *ResoGST52*, a member of the Tau subfamily, as a key gene responsive to drought stress, showing significant drought-induced upregulation in both roots and leaves. Its expression may be regulated by the transcription factor *ResoDof17*, and its co-expression network is enriched in plant hormone signaling and antioxidant metabolism pathways. Evolutionary analysis revealed that *ResoGST52* has undergone positive selection. Mutations at key sites in the protein are predicted to synergistically optimize the enzyme’s catalytic efficiency and functional resilience under drought conditions, potentially by enlarging the substrate-binding pocket and enhancing structural stability. In summary, this research reveals the composition and evolutionary characteristics of the *GST* gene family in *R. soongorica* and elucidates the central role of *ResoGST52* in drought adaptation from multiple perspectives, providing important candidate genes and a theoretical foundation for molecular breeding aimed at improving crop drought tolerance.

## Data Availability

The data are contained within the present article.

## Supplementary Materials

The following supporting information can be downloaded at https://www.mdpi.com/article/10.3390/biology15080660/s1.
