# Supplementary figures and images for: Identification of the GST Gene Family in Reaumuria soongorica and Its Response to Drought Stress
Source: Biology (Basel). 2026 Apr 21;15(8):660. doi: 10.3390/biology15080660 (PMC13113421; doi:10.3390/biology15080660)

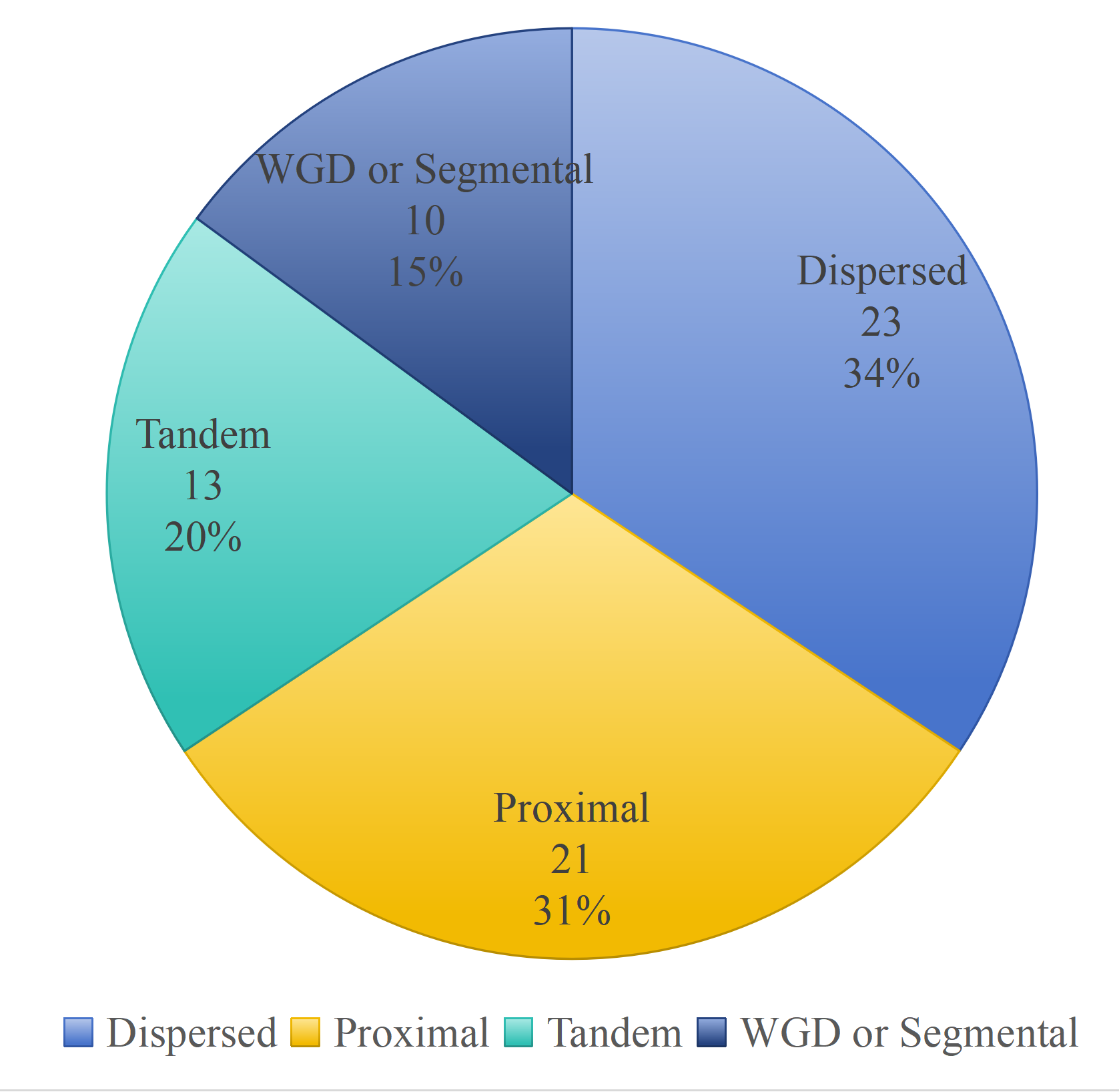

Supplement: Supplementary file 1 [file biology-15-00660-s001.zip › Fig S1.png]

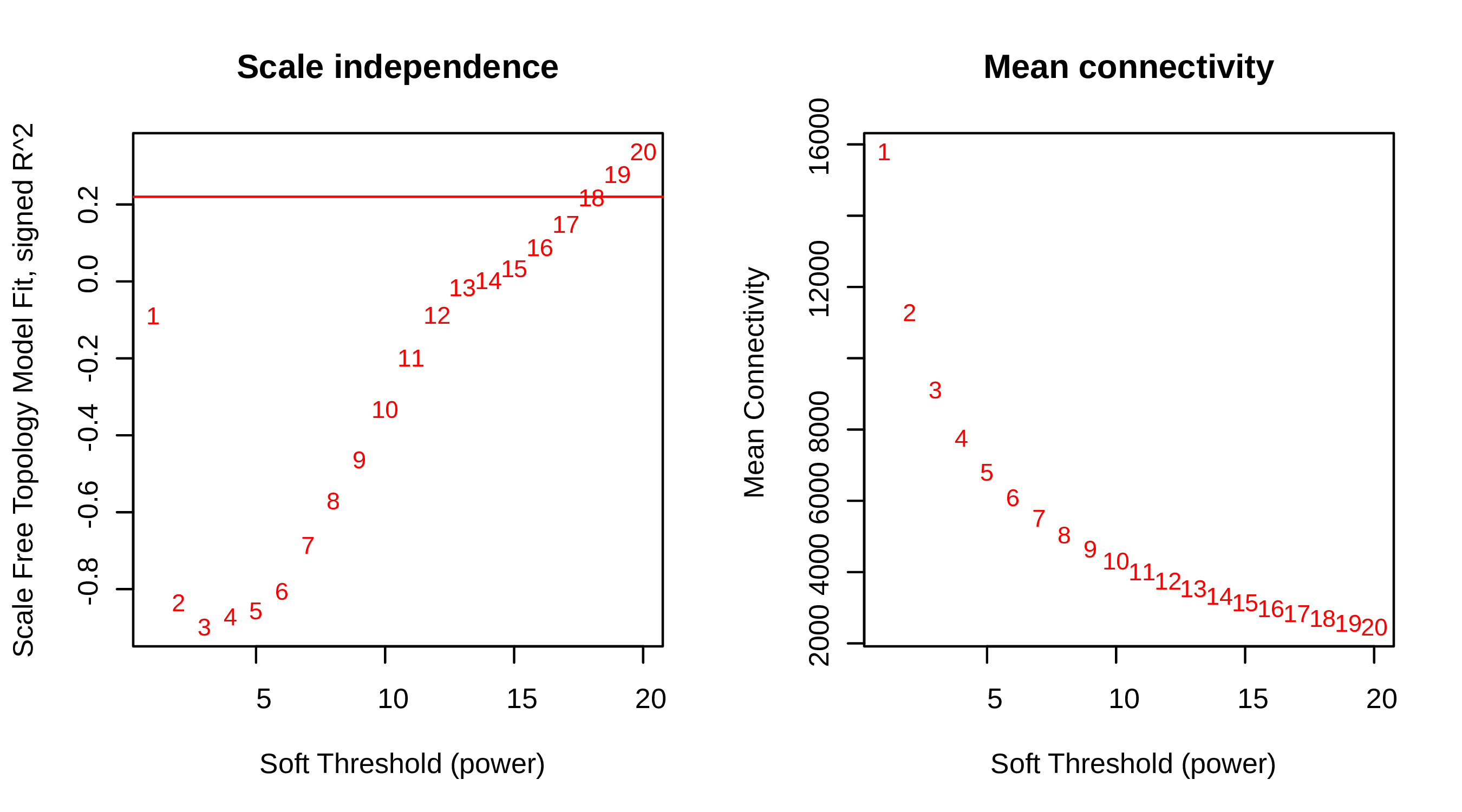

Supplement: Supplementary file 1 [file biology-15-00660-s001.zip › Fig S2.png]
